# Freeze-Drying of Mononuclear Cells Derived from Umbilical Cord Blood Followed by Colony Formation

**DOI:** 10.1371/journal.pone.0005240

**Published:** 2009-04-21

**Authors:** Dity Natan, Arnon Nagler, Amir Arav

**Affiliations:** 1 Core Dynamics Ltd., Ness-Ziona, Israel; 2 Hematology Division, BMT and Cord Blood Bank, Chaim Sheba Medical Center, Israel; 3 Institute of Animal Science, Agricultural Research Organization (ARO), Bet Dagan, Israel; Geroge Mason University, United States of America

## Abstract

**Background:**

We recently showed that freeze-dried cells stored for 3 years at room temperature can direct embryonic development following cloning. However, viability, as evaluated by membrane integrity of the cells after freeze-drying, was very low; and it was mainly the DNA integrity that was preserved. In the present study, we improved the cells' viability and functionality after freeze-drying.

**Methodology/Principal Findings:**

We optimized the conditions of directional freezing, i.e. interface velocity and cell concentration, and we added the antioxidant EGCG to the freezing solution. The study was performed on mononuclear cells (MNCs) derived from human umbilical cord blood. After freeze-drying, we tested the viability, number of CD34^+^-presenting cells and ability of the rehydrated hematopoietic stem cells to differentiate into different blood cells in culture. The viability of the MNCs after freeze-drying and rehydration with pure water was 88%–91%. The total number of CD34^+^-presenting cells and the number of colonies did not change significantly when evaluated before freezing, after freeze-thawing, and after freeze-drying (5.4×10^4^±4.7, 3.49×10^4^±6 and 6.31×10^4^±12.27 cells, respectively, and 31±25.15, 47±45.8 and 23.44±13.3 colonies, respectively).

**Conclusions:**

This is the first report of nucleated cells which have been dried and then rehydrated with double-distilled water remaining viable, and of hematopoietic stem cells retaining their ability to differentiate into different blood cells.

## Introduction

Cryopreservation of hematopoietic stem cells (HSC) is the backbone of clinical stem cell transplantation (SCT), and this technique is an essential part of autologous SCT [1], cord blood transplantation (CBT) and in many cases, allogeneic SCT [2], particularly when the donor is elderly and the stem cells are not readily available. Moreover, in haplo-identical transplants, the donor undergoes multiple stem cell mobilization and harvesting, which necessitates cryopreservation of the graft [3].

Hematopoietic as well as other somatic cells are currently cryopreserved and stored in liquid nitrogen (LN) tanks, in nitrogen vapor phase or in −80°C freezers [4], [5], [6]. This conventional mode of cryopreservation is prone to transient warming events and various other hazards, such as cross-contamination [7], [8], and results in cell losses of 20 to 30% [9]. In contrast, freeze-drying (i.e. lyophilization) of the cells should theoretically substantially decrease the risks associated with storage at ultra-low temperatures, thus simplifying the procedure, reducing costs and allowing for better management of sample storage and transport. These advantages are especially important when long-term preservation is required, e.g. for potential use at an advanced age. In addition, freeze-drying does not involve the thawing process to which cell damage is attributed, particularly at large volumes (e.g. from recrystallization).

In order to freeze-dry the cells, we first had to develop a method that would enable freezing in the absence of permeating cryoprotectant agents (CPAs) such as DMSO, glycerol or ethylene glycol, to name a few, and would enable the use of additives that have a high glass transition temperature (Tg) and that are solid at room temperature. Overcoming these obstacles is not simple. As Thomas A. Jennings said “most investigators have at times overlooked the importance of the freezing process…while simple in concept, the freezing process will be shown to be perhaps the most complex and least understood step in the lyophilization process” [10]. The major damaging factors associated with freeze-drying liposomes are lipid-phase transition (LPT) and fusion [11]. Cellular membrane LPT is also the mechanism underlying damage that occurs during chilling and it is the main obstacle for successful cryopreservation of many cell types, including sperm [12] and oocytes [13]. However, cryopreservation of cells which are more complex then liposomes has even more damage mechanisms, on top of those to the membrane lipids, such as damage to cells structure and cytoskeleton during freezing [14], [15], damage to lysosomes and mitochondria when cells are dehydrated during the freezing process [16]. cells desiccation causes down-regulation of metabolism, increase intracellular viscosity and salt concentrations, denaturation of proteins and the creation of free radicals [17]–[20]. Chilling injury can be overcome by stabilizing the membrane phospholipids using disaccharides such as sucrose or trehalose [21]. Other approaches to decreasing the damage associated with LPT have involved changing the lipid composition of the membrane by using liposomes in vitro [22] or dietary additives in vivo [23]. Altering membrane lipid composition has been shown to improve the freeze-drying of platelets [24]. The second factor is membrane fusion, which can occur when the dried cells, maintained in a fluid matrix, come into contact [25]. Liposomes stored above the Tg have been shown to rapidly fuse and become damaged, and it was therefore concluded that glass transition or vitrification is an important factor in decreasing the chances of fusion upon drying [26]. Vitrification is normally achieved by combining a high concentration of CPA (high-viscosity), a rapid cooling rate and a small volume (i.e. 0.5 µl) [27], [28]. Obviously, these conditions are not feasible in the freeze-drying of many cell types because of the need to achieve a stable glass matrix without CPAs, at relatively slow cooling rates and large volumes.

We have recently shown that freeze-dried cells can direct embryonic development following cloning [29]. However, those cells had poor viability, showing mainly nuclear integrity. Here we describe the further development of our freeze-drying technology, resulting in improved cell viability and functionality.

We developed a freezing technique in which a thermal gradient is maintained in a conductive material and the sample to be frozen is moved at a controlled velocity through this gradient. After seeding is performed at the edge of the sample, ice crystals start to propagate at a velocity which is correlated to the velocity at which the sample passes through a predetermined thermal gradient. Cooling rate, calculated as thermal gradient (G) multiplied by velocity (V), can be precisely controlled [30], [31]. Using this method, we improved the outcome of freezing bulk samples, such as large volumes of sperm [31]–[33] and whole organs [34], [35]. In addition, this method was used by others and found superior to conventional equiaxial freezing [36], [37].

Epigallocatechin gallate (EGCG) is a polyphenol found naturally in green tea (Folium Camelliae). EGCG is a potent antioxidant, with a wide range of physiological effects, including anticarcinogenic, antibacterial, and antiviral properties [38]–[40] and, of course, it reduces oxidative stress [41]. Furthermore, we recently found that EGCG also reduces heat stress in mice [42]. We used EGCG as a new additive in our cryopreservation solution. Apparently due to EGCG's interaction with the cell membrane [43] and its antioxidative properties, it stabilized the cells during lyophilization and storage.

In the present study, we evaluated the ability to freeze-dry mononuclear cells (MNCs) derived from human umbilical cord blood (HUCB).

## Materials and Methods

### Blood collection and separation

HUCB units were received from mothers who gave their written consent, under approval of the Helsinki committee—approval no. 1341/01, at the “Chaim Sheba Tel Hashomer” Cord Blood Bank.

MNCs were separated on a Ficoll-Paque density gradient. Briefly, 3 ml of whole blood was carefully layered above 3 ml of Histopaque-1077 (Sigma, St. Louise, MO, USA) and centrifuged for 30 min at 1000 *g* (with no break), after which the MNC layer was withdrawn, placed into another test tube with 10 ml of phosphate buffered saline (PBS; free of Ca^2+^ and Mg^2+^) and centrifuged for 10 min at 250 *g*. After this centrifugation, the supernatant was discarded, and a freezing solution at room temperature was added to the cell pellet. The volume of the freezing solution varied, depending on the desired cell concentration (we experimented with concentrations ranging from 1 to 10×10^6^ cells/ml). A 2.5-ml cell suspension was transferred into a 16-mm-diameter glass test tube (Manara, Kibuts Manara, Israel) which was then frozen in an MTG-1314 apparatus (Core Dynamics, Nes-Ziona, Israel). Samples were placed in the refrigerator for 10–39 minutes before being put in the MTG device for the freezing process.

### Freezing solution

Our cryopreservation solutions were based on Ca^2+^- and Mg^2+^-free PBS supplemented with one or more of the following additives: (1) 0.1 M trehalose (Sigma), (2) 12.5% (w/v) human serum albumin (HSA; Kamada, Beit-Kama, Israel), (3) epigallocatechin gallate (EGCG) (>98% purity; Zhejiang Yixin Pharmaceutical, Lanxi, China), (4) dextran 40 (Pharmacosmos, Holbaek, Denmark). For most of the experiments, we settled on IMT-2 solution, composed of 0.945 mg/ml EGCG and 0.1 M trehalose dissolved in Ca^2+^- and Mg^2+^-free PBS.

### Freezing and drying

Freezing was performed in the MTG-1314 apparatus. The freezing-machine temperatures were set to: 5°C to −10°C to −40°C to −70°C. Seeding was performed at the tip of the test tube as the samples entered the cold block (at −10°C). The velocity at which the samples were pushed through the freezing machine was set at 0.2 mm/s, resulting in a calculated cooling rate of 5.1°C/min; the actual cooling rate, as measured by placing thermocouples in a sample, was 4.05°C/min. After completion of the freezing process, samples were stored in a LN tank. Samples were then either thawed or moved on to the drying process which was performed in a commercial lyophilizer (Labconco, Kansas City, MO, USA).

Lyophilization was performed by putting the frozen samples in the commercial lyophilizer near the condenser (Freezone Plus 6, Labconco), which reaches a temperature of −80°C, for 3.5 days. This freeze-drying system is a very simple device, in which neither shelf temperature nor vacuum pressure can be controlled or recorded.

### Thawing, rehydration, and storage

Thawing was performed by immersing the frozen samples in a water bath heated to 37°C. At the end of the lyophilization process, samples were either immediately rehydrated or stored, the latter by putting the samples within an aluminum pouch under vacuum and heat-sealing the pouch using vacuum chamber packaging machine Audionvac VMS 163 (Audion Elektro, Weesp, The Netherlands). The samples were then stored in a refrigerator (2°C–8°C) or at room temperature (25°C) for up to 1 week. Rehydration was performed by adding 2.4 ml double-distilled water (DDW) that had been pre-warmed to 37°C.

After both thawing and rehydration, samples were assessed for viability by Syto-13/PI staining, for CD34^+^-presenting cells by fluorescence-activated cell sorting (FACS) and for colony-forming units (CFU) by assay.

All assessments were performed before and after cryopreservation.

### Residual moisture content and Tg measurements

Three samples from three different donors were evaluated following freeze-drying for their residual moisture content and for glass transition temperature (Tg). For the Tg evaluations, samples were sent to the Analytical Research Services and Instruments unit of Ben-Gurion University where the measurement was performed using a differential scanning calorimeter (DSC) 821e (Mettler, Toledo, OH). The samples were heated from −80°C to 50°C at 10 K/min, blank curve corrected, in a nitrogen atmosphere (80 cm^3^/min).

For the residual moisture measurement, we used an HB43 halogen moisture analyzer (Mettler). In brief, samples in powdered form following the lyophilization process are heated up in the moisture analyzer. During the heating process, the analyzer weighs the sample for a pre-determined time, and the measurement is recorded as weight loss of the sample with time.

### Viability and recovery

Cell viability was assessed using the live/dead fluorescent stains Syto-13/PI (Molecular Probes®, Invitrogen Crop., Carlsbad, CA, USA) for membrane integrity. The percentage of viable cells was calculated as follows:
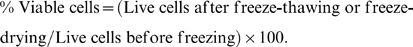
Cell recovery was evaluated using the automatic cell counter Pentra 60 (Horiba ABX, Montpellier, France). Percent cell recovery was determined as follows:
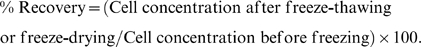



### Surface antigens

We examined the MNCs from each HUCB unit before and after freeze-thawing or freeze-drying for number of cells presenting CD34^+^ antigen. Briefly, 1×10^6^ MNCs were stained with 1 µg fluorescein isothiocyanate (FITC)- or phycoerythrin (PE)-conjugated monoclonal antibodies for 20 min at 4°C and then washed in PBS containing 0.02% (w/v) azide and 1% (v/v) bovine serum albumin as described previously [44]. The cells were stored at 4°C in 0.5 ml of 1% (v/v) paraformaldehyde until analysis. Fluorescence intensity was measured on a FACScan (Becton Dickinson, Franklin Lakes, NJ, USA).

### Colony-forming unit assay

CFU assay was performed on MNCs derived from each HUCB unit using fresh samples (i.e. samples exposed to the IMT-2 solution but not frozen) and samples after freeze-thawing or freeze-drying.

We used the protocol and Methocult media from Stem Cells Technologies Inc. (Vancouver, Canada). Briefly, cells were inoculated into methylcellulose medium and incubated in 5% CO_2_ for 14 days. After incubation, the plate was viewed under an inverted microscope, and cell colonies were counted and identified as erythrocytic (CFU-E, BFU-E), granulocytic (CFU-GM), or mixed (CFU-GEMM).

### Scanning electron microscopy

For evaluation of the morphology of dry samples Scanning Electron Microscopy (SEM) was used. MNC derived from HUCB were frozen in three different solutions: (1) trehalose solution – 0.1 M trehalose in PBS (without Ca^+2^ & Mg^+2^). (2) EGCG solution – 0.945 mg/ml EGCG dissolved in PBS (without Ca^+2^ & Mg^+2^). (3) IMT-2. The samples were frozen and dried as described above. After lyophilization has finished samples were gold plated before being placed in the SEM. The voltage of the electron scatter was 25 kv. In this test the electrons hit the sample and are returned to a detector. We captured a 3D picture on a CRT screen.

### Statistical analysis

At least three replications were performed for each experiment (i.e., using blood from three different donors). At least 300 cells were counted per sample. Means were calculated and differences between treatments were examined by t-tests using the General Linear Model procedure of JMP (SAS Institute, 1994, Cary, NC). Significance was *P*<0.05 unless otherwise stated. In all figures, different letters represent statistically different samples. Results are reported as mean±SE.

## Results

### Moisture content and Tg

After freeze-drying, samples were taken out of the lyophilizer and analyzed in the HB43 halogen moisture analyzer. The results showed a residual moisture content of 4.69±0.07% in all samples tested (all samples were frozen in IMT-2 solution). The Tg of the dried samples was between 8.49°C and 25.94°C, with a midpoint at 11.84°C.

### Effect of ice interface velocity on survival after freeze-drying

We evaluated the survival of HUCB MNCs after freeze-thawing and freeze-drying at different cooling rates, resulting from different ice interface velocities (V). The freezing solution consisted of 12.5% (w/v) HSA and 0.1 M trehalose in Ca^2+^- and Mg^2+^-free PBS.

The freezing temperatures in the MTG-1314 were set as described in Materials and Methods. The different cooling rates were achieved by varying the velocity at which the samples were pushed through the predetermined temperatures. The ice interface velocities were: 0.02, 0.2 and 2 mm/s, resulting in calculated cooling rates of 0.51, 5.1 and 51°C/min, respectively (cooling rate = G×V).

**Figure 1:**
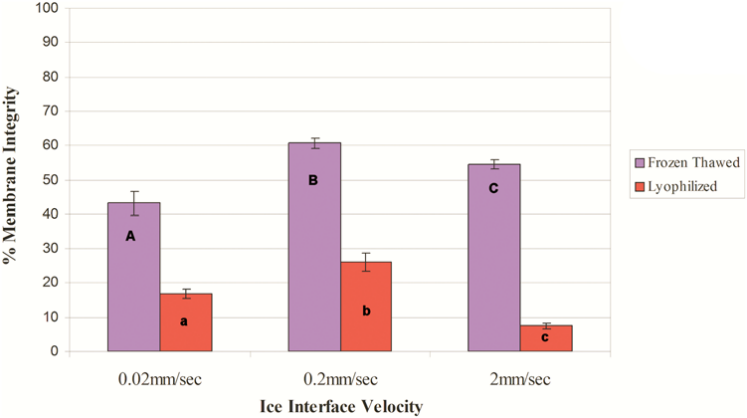
Percentage membrane integrity after freezing at different ice interface velocities. The solution used in this experiment was 12.5% (w/v) HSA and 0.1 M trehalose in PBS (Ca^2+^- and Mg^2+^-free). This experiment was performed on HUCB units from three different donors, in triplicate (n = 54). Data are presented as mean±SE.

Figure 1 depicts the effect of ice interface velocity on cell survival, according to fluorescent Syto/PI staining after freeze-thawing and freeze-drying. In both cases, the highest viability rates were obtained at the intermediate velocity of 0.2 mm/s, resulting in 60% and 25% of the cells having intact membranes after freeze-thawing and freeze-drying, respectively.

### Effect of different additives on cell survival after freeze-drying

We evaluated the survival of the MNCs after freeze-thawing and freeze-drying in freezing solutions with different viscosities. All other experimental steps were performed as described in Materials and Methods.

**Figure 2:**
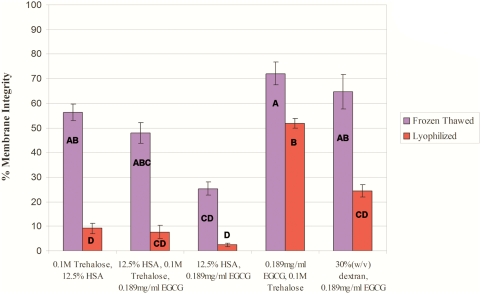
Percentage membrane integrity after freeze-thawing and freeze-drying in different solutions. All solutions are based on PBS (Ca^2+^- and Mg^2+^-free). This experiment was performed on HUCB units from three different donors, in duplicate (n = 60). Data are presented as mean±SE. donors, in duplicate (n = 60). Data are presented as mean±SE.

Figure 2 presents the effects of different cryopreservation solutions on HUCB-derived MNC viability after freeze-thawing and freeze-drying. The original solution used for the preliminary studies, composed of HSA and trehalose (solution #1), produced rather low viability rates, especially after freeze-drying (only 10%). However, the solution consisting of trehalose and EGCG (solution #4) and that consisting of EGCG and dextran (solution #5) produced higher viability rates, resulting in 70% and 52% of the cells having intact membranes after freeze-thawing and freeze-drying, respectively, for solution #4, and 65% and 25% of the cells having intact membranes after freeze-thawing and freeze-drying, respectively, for solution #5.

We compared cell recovery in the three solutions that gave the highest viability rates. The HSA-trehalose solution yielded recovery rates of 100% and 85% after freeze-thawing and freeze-drying, respectively. However, this solution showed a low viability rate after freeze-drying (10% membrane integrity, see Figure 2). The dextran-trehalose solution yielded recovery rates of 98% and 62% after freeze-thawing and freeze-drying, respectively. This solution showed a rather large reduction in cell recovery after freeze-drying. Combined with a low viability of 25%, only 15% of the cells were actually viable. The trehalose-EGCG solution resulted in 100% recovery after both freeze-thawing and freeze-drying.

### Dose response of EGCG

We evaluated the effect of different EGCG concentrations on HUCB-derived MNC survival following freeze-thawing and freeze-drying.

**Figure 3:**
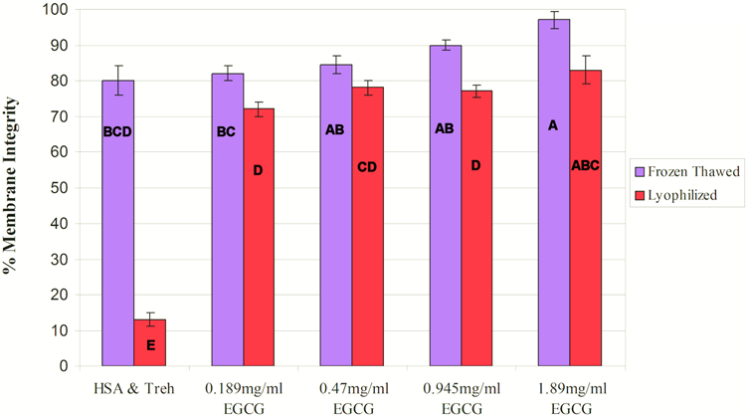
Percentage membrane integrity after freeze-thawing and freeze-drying with different concentrations of EGCG. This experiment was performed on HUCB units from three different donors, in duplicate (n = 60). Data are presented as mean±SE.

The freezing solutions were composed of 0.1 M trehalose and EGCG at the following concentrations: 0.189, 0.47, 0.945 or 1.89 mg/ml, all in Ca^2+^- and Mg^2+^-free PBS. Results are presented in Figure 3. The highest concentration of EGCG gave the best viability rates after freeze-thawing and freeze-drying, with 97.9% and 88% of the cells having intact membranes, respectively. However, antioxidants are known to have a damaging effect at high doses. Therefore, we used the lower dose of 0.945 mg EGCG/ml (which, with 0.1 M trehalose in Ca^2+^- and Mg^2+^-free PBS, makes up IMT-2) in further experiments, as this dose also resulted in very high viabilities—82.8% and 77% of the cells with an intact membrane after freeze-thawing and freeze-drying, respectively. The recoveries in this experiment were more then 98% in all samples, therefore, only viability data is shown since we remained with the same cell number after freeze thawing and freeze drying.

### Effect of each freezing solution components on post-thaw and post-rehydration viability and the influence of a washing process after these procedures

In this set of experiments we wanted to find out how each of the ingredients composing IMT-2 freezing solution (i.e. EGCG and trehalose) affect the post thaw and post rehydration viability of MNC derived from HUCB. In addition, we also wanted to find out how a washing procedure after thawing and rehydration will affect the viability.

These experiments were done on UCB form 4 different donors. The MNC were suspended with one of the following freezing solutions:

1. IMT-2
2. PBS (without Ca^++^ & Mg^++^) supplemented with 0.945 mg/ml EGCG.
3. PBS (without Ca^++^ & Mg^++^) supplemented with 0.1 M Trehalose

From each unit a total of 12 samples were frozen using the MTG as described above, half of the samples (n = 6) continued to the drying process. The experiment was performed with duplicates for each solution. Rehydration and thawing were performed as described above. Immediately after thawing and rehydration a samples was taken to determine cells concentration and viability, the rest of each sample (1.5 ml) underwent a washing process by centrifuging it for 10 minutes at 250 g after which the supernatant was discarded and the pellets were re-suspended with 1.5 ml RPMI-1640 (Sigma, St. Louise, MO, USA).

**Figure 4:**
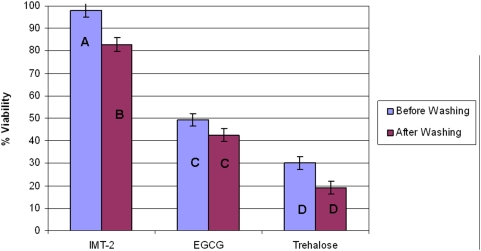
Viability percentage of MNC derived from HUCB after freeze thawing with one of the following solutions: IMT-2, EGCG solution, Trehalose solution. The samples were either immediately evaluated or underwent a washing process and then evaluated again. The data is presented as the mean±SE, different letters represent statistical differences between groups (p<0.05). The means were calculated as the number of viable cells after thawing divided by the number of viable cells before freezing. (N = 48).

In this experiment we calculated the viability after each procedure according to the following formula:
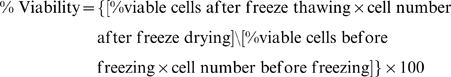
The results after freeze thawing were as follows: for IMT-2 solution 98.01%±3.1% immediately after thawing and 82.75%±3.1% after washing; for the EGCG solution viability after thawing was 49.27%±2.9% and after washing it was 42.47%±2.9%; the EGCG solution resulted with a viability of 30.1%±2.9% after freeze thawing and 19.18%±2.9% after thawing and washing (Figure 4).

**Figure 5:**
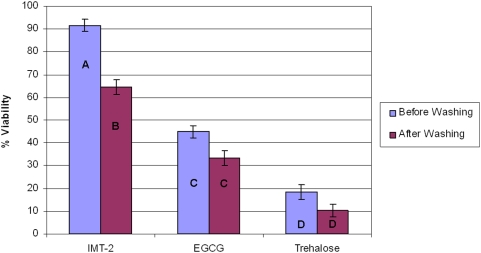
Viability percentage of MNC derived from HUCB after freeze drying with one of the following solutions: IMT-2, EGCG solution, Trehalose solution. The samples were either immediately evaluated or underwent a washing process and then evaluated again. The data is presented as the mean±SE, different letters represent statistical differences between groups (p<0.05). The means were calculated as the number of viable cells after thawing divided by the number of viable cells before freezing. (N = 48).

The results after freeze drying and rehydration were as follows: for IMT-2 solution 91.6%±2.6% immediately after rehydration and 64.55%±3.1% after washing; for the EGCG solution viability after rehydration was 44.8%±2.7% and after washing it was 33.38%±3.1%; the EGCG solution resulted with a viability of 18.52%±2.6% after rehydration and 10.28%±3.1% after washing (Figure 5). The data is presented as mean±SE, (n = 48 for each cryopreservation procedure).

We can see that EGCG has a higher contribution to the post thaw and post rehydration viability of the cells then Trehalose (Figures 4 and 5). Centrifugation has resulted with a reduction in cell viability but only with IMT-2 solution the differences were statistically significant (Figures 4 and 5).

### Effect of different cell concentrations on their survival after freeze-drying

We evaluated whether cell concentration affects post-lyophilization viability. We tested the following cell concentrations: 2.5×10^6^±0.4×10^6^ cells/ml, 5×10^6^±0.3×10^6^ cells/ml and 8×10^6^±0.2×10^6^ cells/ml. All samples were frozen in IMT-2 solution, and freezing and drying were performed as described in Materials and Methods. This experiment was performed on HUCB units from three different donors, in triplicate (n = 18). Membrane integrity as assayed by Syto/PI staining was as follows: 69.28±17.1%, 57.01±14.1% and 55.9±5.3% for the cell concentrations listed above, respectively. Thus there was a reduction in viability with increasing cell concentration; however, the differences were not statistically significant.

### Effect of storage conditions on cell viability

In these experiments, freeze-dried samples of HUCB-derived MNCs were stored in the dry state at two temperatures: 4°C (refrigeration) and room temperature. Samples were stored for 3 days and for 1 week, after which they were rehydrated with DDW and their viability evaluated by Syto/PI staining. All samples were stored under vacuum and protected from the light. The results were as follows: after 3 days of storage at 4°C, membrane integrity was 52.5±12% and after 1 week at 4°C it was 72.1±11.5%. After 3 days of storage at room temperature, membrane integrity was 50±10% and after 1 week at room temperature it was 50.5±10%. This experiment was performed on HUCB units from three different donors, in triplicate (n = 36). There was no statistical difference between the tested groups.

### Number of CD34^+^presenting cells before and after freeze-thawing and freeze-drying

In these experiments, MNCs that had been frozen in IMT-2 solution were evaluated for CD34^+^ cells before and after freeze-thawing and freeze-drying. From each unit of HUCB, at least four test tubes were frozen and subjected to either thawing or drying and rehydration. Cell counts are presented in Table 1.

**Table 1:** Number of CD34+ cells before freezing and after thawing and lyophilization.

Exp #	1	2	3	
**Fresh (×10^4^ cells)**				
Group 1	10.4	1.05	4.85	
**Mean±SD**				**5.4±4.7**
**After thawing (×10^4^ cells)**				
Group 1	1.9	15.6	0.7	
Group 2	2.3	0.25	0.2	
**Mean±SD**				**3.5±6**
**After rehydration (×10^4^ cells)**				
Group 1	1.6	36.4	1.7	
Group 2	------	0.2	2.9	
Group 3	5.7	2	0.15	
**Mean±SD**				**6.3 ±12.3**

Results are from three different experiments (i.e. different donors); each individual number represents a different sample.

### Colony-forming unit assay

In these experiments, the same MNC samples used for CD34^+^ cell detection were subjected to CFU assay before and after freeze-thawing or freeze-drying and rehydration. The colonies were evaluated and the total number of colonies for each unit is shown in Table 2.

**Table 2:** The number of colonies grown, before freezing and after thawing and rehydration.

Exp #	1	2	3	
**Fresh**				
Group 1	**60**	**18**	**15**	
	(33-E/BFU; 27-GM)	(6-E/BFU; 12-GM)	(5-E/BFU; 10-GM)	
**Mean±SD**				**31±25.15**
**After thawing**				
Group 1	**12**	**21**	**10**	
	(4-E/BFU; 8-GM)	(3-E/BFU; 18-GM)	(GM)	
Group 2	**53**	**29**	**108**	
	(31-E/BFU; 22-GM)	(8-E/BFU; 21-GM)	(1-E/BFU; 107-GM)	
**Mean±SD**				**38.83±37.3**
**After rehydration**				
Group 1	**9**	**42**	**27**	
	(2-E/BFU; 7-GM)	(11-E/BFU; 31-GM)	(3-E/BFU; 24-GM)	
Group 2	**30**	**28**	**12**	
	(1-E/BFU; 29-GM)	(1-E/BFU; 27-GM)	(GM)	
Group 3	**13**	**43**	**6**	
	(1-E/BFU; 12-GM)	(4-E/BFU; 39-GM)	(GM)	
**Mean±SD**				**23.3±13.9**

Results are from three different experiments (i.e. different donors); each individual number represents a different sample and the colony type is given in brackets (erythrocytic- E/BFU; granulocytic – GM).

## Discussion

The freeze-drying of cells can be divided into two steps: the first is the freezing process during which large ice crystals are formed, pushing the cells into an area defined as the unfrozen fraction. The unfrozen fraction incorporates the cells that have dehydrated and vitrified in an amorphous matrix during the freezing process. The second step is sublimation of the ice crystals, which occurs in two stages, termed primary and secondary drying. Two requirements for cell survival during freeze-drying are stabilization of the membrane during the drying process [11] and the ability to form a stable vitrified matrix of cells in the unfrozen fraction. Stabilization of the membrane during dehydration is important for both the freeze-drying process and for room-temperature storage of the dried material [25]. Crowe et al. [45], [46] have shown that both of these requirements can be fulfilled by using the non-reducing disaccharide trehalose, which is present in many organisms (at up to 20% of their dry weight) that dehydrate almost completely in nature. Intracellular trehalose has been shown to be necessary for successful stabilization of the membrane during freeze-drying of liposomes and cells [47]. In the present study, we show that optimizing the freezing process and providing extracellular trehalose and EGCG is sufficient for successful freeze-drying of MNCs.

When we evaluated the effect of ice interface velocity (Figure 1) on the viability of freeze-thawed and freeze-dried HUCB-derived MNCs, we obtained an inverted U-shaped viability curve in which the highest survival rate was achieved at the intermediate velocity of 0.2 mm/s. We used an MTG-1314 freezing apparatus, which is based on directional freezing. With the MTG, cooling rate depends on the ice interface velocity (V) and the thermal gradient (G). Interface velocity is known to affect the survival of cells after freeze-drying via its control of the size and morphology of the ice crystals [30]. At a slow ice interface velocity (0.02 mm/s), large ice crystals are formed, minimizing the size of the unfrozen fraction, and thus causing mechanical damage to the cells and cell-shearing. On the other hand, at the high ice interface velocity (2 mm/s), the size of the unfrozen fraction is large but the likelihood of intracellular ice formation increases, since water has less time to diffuse out of the cells during the freezing process [48]. Further examination indicated that our original freezing solution (composed of HSA and trehalose) was not optimal, and that the solution that included EGCG and trehalose was better suited to our purposes (Figure 2). EGCG is an antioxidant which is also known to connect to the polar head groups of membrane lipids [43] and recently it has been shown that EGCG penetrate suspended cells in a time dependant manner [50]. We hypothesized that this ability of EGCG stabilized the membrane during the freeze-thawing and freeze-drying processes; this stabilization was effected in a dose-related manner (Figure 3). This was also shown in the experiments were we have evaluated the effect of each solution additive on the post thaw and post rehydration viability. The results in Figures 4 and 5 demonstrate that the effect EGCG has on the viability of cells is higher then that of Trehalose and that having both of the components in the solution (i.e. IMT-2) has resulted with very high viabilities after freeze thawing and freeze drying. In addition, the viabilities received after freeze drying for IMT-2 and EGCG solution were similar to the viabilities received with these solutions after freeze thawing. Whereas with trehalose there was a much lower viability after freeze drying then after freeze thawing. In our method we do not introduce trehalose into the cells. It has been described that the having intracellular trehalose is of importance in stabilizing membranes for freeze drying [47]. The EGCG ingredient which results with similar viabilities after both procedures imposes sufficient protection on the cells during both freeze thawing and freeze drying. In addition, it seems that both supplements have a synergetic effect since the viability obtained with IMT-2 solution is higher then the sum of each component combined. It has been demonstrated that a stress protein acts synergistically with trehalose to confer desiccation tolerance [49].

**Figure 6:**
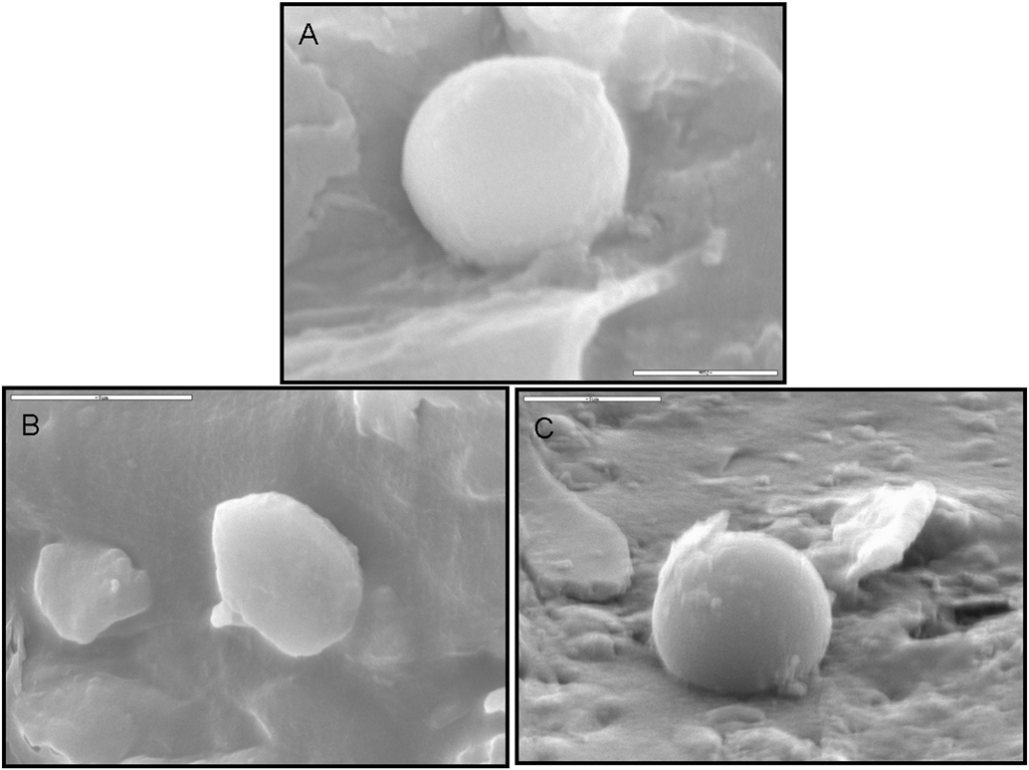
Pictures of taken using a scanning electron microscope of freeze-dried MNC derived from HUCB. The pictures where of samples frozen with one of the following solutions: (A) Cells that were frozen with 0.1 M trehalose in PBS (sans calcium & magnesium). (B) Cells that were frozen with 0.945 mg/ml EGCG in PBS (sans calcium & magnesium). (C) Cells that were frozen with IMT-2 solution. Pictures Magnification ×7500.

As for the effect washing procedure has on the viability; we can see that washing has resulted with a reduction in viability with the IMT-2 solution both after freeze thawing and both after freeze drying, indicating that although immediate viability was very high (>90% for each procedure) there was a sub-population of cells which were further damaged by the washing procedure. The viability after washing has decreased more after freeze-drying then after freeze thawing, indicating a larger number of cells that were impaired although at first passed as viable cells according to live/dead assay (Figures 4 and 5). When looking at the SEM pictures (Figure 6) we can see that cells that were freeze-dried trehalose solution (Figure 6A) had a good morphology similar to what is seen in cells freeze-dried with IMT-2 solution (Figure 6C). Whereas, the cells freeze-dried with EGCG solution (Figure 6B) were less round and shrunk. When observing the viability results after freeze-drying that each solution gave (Figure 5) EGCG solution had a higher viability percentage then that received with the trehalose solution. This strengthens the idea that trehalose needs to be intracellular as well in order to provide its lyo-protective properties. These experiments indicated the importance of both the size of the unfrozen fraction and membrane stabilization.

Stability of the unfrozen fraction is important during the drying process: if the sublimation temperature is above the Tg of the sample, damage will be incurred due to recrystallization [51]. The cooling rate affects freezing and drying in opposite ways: while it is important that the cooling rate be rapid enough to prevent long exposure to high concentrations of solutes which can cause chemical toxicity and osmotic stress, it should also be slow enough to minimize the chances of intracellular crystallization during freezing. A slow cooling rate is also important for the subsequent drying step, since it allows the growth of large ice crystals, which facilitates sublimation during primary drying [52], [53]. In the freeze-drying process, vitrification (also termed eutectic solidification) occurs in the unfrozen fraction between the ice crystals which are sublimated in an endothermic process. However, Mazur et al. [54] have shown that decreasing the unfrozen fraction can damage the cells, probably due to mechanical damage by the ice crystals. Therefore, the unfrozen fraction should be small enough to allow vitrification but large enough to reduce the damaging effect of the ice crystals. We believe that directional freezing can combine the optimal conditions needed to achieve large unfrozen fractions for a given concentration of cells and additives, because the size of the ice crystals is determined by the interface velocity [30], [31], [55] and is precisely controlled by the rate of movement through the thermal gradient.

It is important that the additive(s) have a high Tg. Moreover, the concentration of the additive is positively correlated with its Tg [56]. Increased solution viscosity will increase the chances of vitrification because at 10^14^ poise, the solution forms a glass [27], [28]. In the present work, we used trehalose as the additive as it has a high Tg combined with membrane-stabilizing properties [57]. We believe that EGCG also gave better results (Figures 2, 3, 4, and 5) than other additives because of its ability to interact with the cell's membrane [43], together with its antioxidant properties [58], [59]. Cell concentration is an important factor during both freezing [60] and drying. Crowe et al. [11] have shown that the primary role of vitrification is to prevent the cells from coming in close proximity in the dry state, thereby preventing fusion and cell damage. Therefore, it can be concluded that cell concentration, unfrozen fraction size and vitrification are important parameters for both the frozen and the dried states, a conclusion that was confirmed in the present study.

The experiments performed with different concentrations of MNCs showed a decrease in viability with increasing cell concentration. A similar observation has been made with erythrocytes and hepatocytes [61]. There are three ways in which cell concentration can influence survival after freeze-drying: (a) During the freezing process, the higher the cell concentration, the higher the chances that more cells will be subjected to mechanical damage (i.e. shearing damage) [60]. (b) During drying, the chances of cell fusion increase with cell concentration. This is because of the increased likelihood of cell membranes coming into contact with each other and fusing, and because a high concentration of cells reduces the Tg. (c) The freezing solution is composed of a certain concentration of additives. When the number of cells exposed to this concentration varies, so does the additive's ability to provide cryoprotection (e.g. by stabilize the membrane). The higher the cells concentration in a pre-fixed volume the fewer individual cells are exposed to the additives in the solution. In the present report, we used IMT-2, made up of trehalose and EGCG, a composition which stabilized the membrane and prevented both fusion and oxidative stress [41], [43], [47].

**Figure 7:**
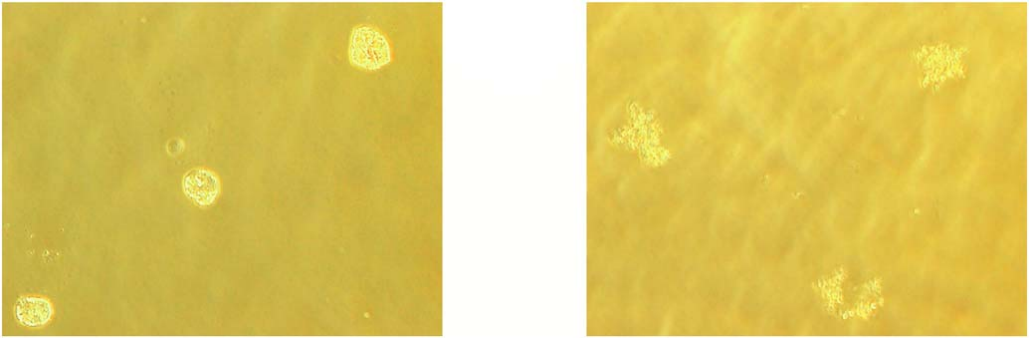
Photographs showing colonies formed after lyophilization and rehydration with DDW of samples frozen in IMT-2 solution.

When we performed the CFU assay on freeze-thawed and freeze-dried and rehydrated MNCs, we observed various types of colonies (granulocytes, macrophages and erythrocytes) after 14 days of culture, indicating, for the first time, that HSCs which have been dried and rehydrated with pure DDW retain their functionality and are able to form colonies of both myeloid and erythroid lineages (Table 2). In addition, we show that after freeze-thawing and freeze-drying and rehydration, cells also retain their CD34^+^ surface antigen (Table 1), a marker for hematopoietic progenitor cells. However, with both the CD34^+^ and colony-formation data, we noted a large variation among samples taken from the same donor and subjected to the same conditions. Although we do not have any explanation for this variation, it has been reported by others as well [62]. In addition, EGCG may have an effect on the clonogenic capacity of the cells. Several effects of EGCG on T cells have been reported, including inhibition of T-cell proliferation and inhibited division of stimulated T cells [63]. In addition, it has been found that EGCG binds to CD4^+^ T cells and it has even been suggested as a way of preventing HIV infection [64]. EGCG has also been found to bind to CD11b and CD8^+^ T cells and to exert strong suppression of CD8^+^ cell migration and adhesion [65]. We theorize that the same mechanism via which EGCG stabilizes cell membranes during freeze-drying is also responsible for the cells' decreased ability to proliferate and differentiate normally via an inhibitory effect. This was evidenced by the number and morphology of the colonies before freezing (and after adding IMT-2 solution) as well as after freeze-drying and rehydration, which yielded a morphology similar to that seen in Figure 7. Nevertheless, certain possibilities for minimizing this damaging effect should be further evaluated, such as removing residual EGCG from the solution by centrifugation after thawing or rehydration, or diluting the EGCG concentration by adding solution—indeed, washing the cells after rehydration increased cell proliferation (data not shown). The effect of EGCG on the cells might be stronger in vitro than when administered in vivo. We have performed experiments with this solution on MNCs derived from male mouse bone marrow which we then transfused into sub-lethally irradiated female mice: in that experiment, we saw an increase in mouse survival. One month after the transfusion, PCR analysis of the transfused mice's blood revealed the presence of the Y chromosome. This work, performed at Hadassah Ein-Kerem Hospital (data not shown), suggested that the cells were capable of incorporation into the bone marrow and of forming new white blood cells. It may be that EGCG binding to the cells is reversed in vivo at the concentration used in the IMT-2 solution. This hypothesis warrants further investigation.

**Figure 8:**
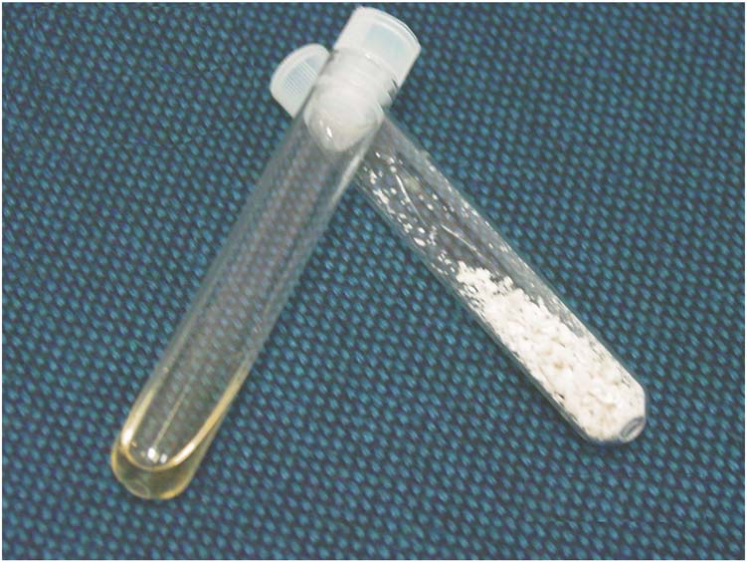
A photo of dry MNCs derived from HUCB after freeze-drying, before (right) and after (left) rehydration with DDW.

We believe that the combination of IMT-2 solution and optimal velocity of ice propagation using directional freezing results in the reduced damage observed here at relatively slow cooling rates and in the absence of CPAs. The reason for the higher viability of cells after freezing relative to post-freeze drying is probably associated with the non-optimal conditions of the drying or storage process. Rindler et al. [66] have shown the importance of sublimation temperature, as recrystallization occurs at a sublimation temperature that can be damaging to the cells. Here we found that maintaining the cells at a temperature of −35°C leads to cell damage and to a loss of membrane integrity (data not shown). In addition, storage at low temperature (4°C) was found to be superior to storage at room temperature. Figure 8 shows photographs of samples after freeze-drying and after rehydration. The sample resembles a powder after freeze-drying (Figure 8), with its relatively low residual moisture content of 4.69±0.07%. At this moisture level, the Tg midpoint was 11.8°C, indicating that the storage temperature needs to be lower than that to maintain cells under the Tg. The results of the Tg measurements performed on the dry samples, showing a Tg midpoint at 11.84°C (ranging from 8.49°C to 25.94°C). The Tg values found here a relatively low compared to observation of Crowe et al [67]. We speculate that the samples may have obtained moisture prior to the Tg measurements. We currently evaluate ways to improve these results. Therefore, with the residual moisture content found here, the samples can be stored under refrigeration (i.e. 2°C–8°C), but not at room temperature.

We are currently working on determining the optimal conditions for sublimation and storage which we believe will result in further increasing the survival of cells after freeze-drying. In addition, we are up-scaling the MTG freezing device to allow for large-volume (between 20 and 150 ml of sample) directional freezing and freeze-drying. Initial experiments have already been performed.

## References

[1] Mounier N, Larghero J, Manson J, Brice P, Madelaine-Chambrin I (2005). Long term hematologic recovery after autologous stem cell transplantation in lymphoma patients: impact of the number of prefreeze and post-thaw CD34+ cells.. Bull Cancer.

[2] Hassan HT, Zeller W, Stockschläder M, Krüger W, Hoffknecht MM, Zander AR (1996). Comparison between bone marrow and G-CSF-mobilized peripheral blood allografts undergoing clinical scale CD34+ cell selection.. Stem Cells.

[3] Aversa F, Tabilio A, Velardi A, Cunnigham I, Terenzi A (1998). Treatment of high risk acute leukemia with T-cell-depleted stem cells from related donors with one fully mismatched HLA haplotype.. N Engl J Med.

[4] Halle P, Tournilhac O, Knopinska-Posluszny W, Kanold J, Gembara P, Boiret N, Rapate C, Berger M, Travade P, Angielski S, Bonhomme J, Deméocq F (2001). Uncontrolled-rate freezing and storage at −80 degrees C, with only 3.5-percent DMSO in cryoprotective solution for 109 autologous peripheral blood progenitor cell transplantations.. Transfusion.

[5] Choi CW, Kim BS, Seo JH, Shin SW, Kim YH, Kim JS (2001). Long term engraftment stability of peripheral blood stem cells cryopreserved using the dump-freezing method in a −80 degrees C mechanical freezer with 10% dimethyl sulfoxide.. Int J Hematol.

[6] Rogers I, Sutherland DR, Holt D, Macpate F, Lains A, Hollowell S (2001). Human UC-blood banking: impact of blood volume, cell separation and cryopreservation on leukocyte and CD34(+) cell recovery.. Cytotherapy.

[7] Bielanski A, Bergeron H, Lau PC, Devenish J (2003). Microbial contamination of embryos and semen during long term banking in liquid nitrogen.. Cryobiology.

[8] Morris GJ (2005). The origin, ultrastructure, and microbiology of the sediment accumulating in liquid nitrogen storage.. Cryobiology.

[9] Laroche V, McKenna DH, Moroff G, Schierman T, Kadidlo D, McCulloogh J (2005). Cell loss and recovery in umbilical cord blood processing: a comparison of postthaw and postwash samples.. Transfusion.

[10] Jennings TA (2002). Lyophilization.

[11] Crowe JH, Oliver AE, Hoekstra FA, Crowe LM (1997). Stabilization of dry membranes by mixtures of hydroxyethyl starch and glucose: the role of vitrification.. Cryobiology.

[12] Drobnis EZ, Crowe LM, Berger T, Anchordoguy TJ, Overstreet JW, Crowe JH (1993). Cold shock damage is due to lipid phase transitions in cell membranes: a demonstration using sperm as a model.. J Exp Zool.

[13] Arav A, Zeron Y, Leslie SB, Behboodi E, Anderson GB, Crowe JH (1996). Phase transition temperature and chilling sensitivity of bovine oocytes.. Cryobiology.

[14] Weiss L, Armstrong JA (1959). Structural changes in mammalian cells associated with cooling.. Biophysic and Biochem Cytol.

[15] Hosu BG, Mullen SF, Critser JK, Forgacs G (2008). Reversible Disassembly of the Actin Cytoskeleton Improves the Survival Rate and Developmental Competence of Cryopreserved Mouse Oocytes.. PLoS ONE.

[16] McGann LE, Yang H, Walterson M (1988). Manifestations of cell damage after freezing and thawing.. Cryobiology.

[17] Potts M, Slaughter SM, Hunneke FU, Garst JF, Helm RF (2005). Desiccation Tolerance of Prokaryotes: Application of Principles to Human Cells.. Integr Comp Biol.

[18] Potts M (1994). Desiccation tolerance of prokaryotes.. Microbiol Rev.

[19] Wang W (2000). Lyophilization and development of solid protein pharmaceuticals.. Int J Pharm.

[20] Allison SD, Randolph TW, Manning MC, Middleton K, Davis A, Carpenter JF (1998). Effects of drying methods and additives on structure and function of actin: mechanisms of dehydration-induced damage and its inhibition.. Arch Biochem Biophys.

[21] Crowe LM, Crowe JH (1991). Solution effects on the thermotropic phase transition of unilamellar liposomes.. Biochim Biophys Acta.

[22] Zeron Y, Tomczak M, Crowe J, Arav A (2002). The effect of liposomes on thermotropic membrane phase transitions of bovine spermatozoa and oocytes: implications for reducing chilling sensitivity.. Cryobiology.

[23] Zeron Y, Sklan D, Arav A (2002). Effect of polyunsaturated fatty acid supplementation on biophysical parameters and chilling sensitivity of ewe oocytes.. Mol Reprod Dev.

[24] Leidy C, Gousset K, Ricker J, Wolkers WF, Tsvetkova NM (2004). Lipid phase behavior and stabilization of domains in membranes of platelets.. Cell Biochem Biophys.

[25] Sun WQ, Leopold AC, Crowe LM, Crowe JH (1996). Stability of dry liposomes in sugar glasses.. Biophys J.

[26] Crowe JH, Carpenter JF, Crowe LM (1998). The role of vitrification in anhydrobiosis.. Annu Rev Physiol.

[27] Arav A (1992). Vitrification of oocytes and embryos.. Embryonic Development and Manipulation in Animal Production.

[28] Fahy GM, Levy DI, Ali SE (1987). Some emerging principles underlying the physical properties, biological actions, and utility of vitrification solutions.. Cryobiology.

[29] Loi P, Matsukawa K, Ptak G, Clinton M, Fulka J (2008). Freeze-dried somatic cells direct embryonic development after nuclear transfer.. PLoS ONE.

[30] Arav A, Yavin S, Zeron Y, Natan D, Dekel I, Gacitua H (2002). New trends in gamete's cryopreservation.. Mol Cell Endocrinol.

[31] Arav A, Zeron Y, Shturman H, Gacitua H (2002). Successful pregnancies in cows following double freezing of a large volume of semen.. Reprod Nutr Dev.

[32] Gacitua H, Arav A (2005). Successful pregnancies with directional freezing of large volume buck semen.. Theriogenology.

[33] Saragusty J, Gacitua H, King R, Arav A (2006). Post-mortem semen cryopreservation and characterization in two different endangered gazelle species (Gazella gazella and Gazella dorcas) and one subspecies (Gazella gazelle acaiae).. Theriogenology.

[34] Arav A, Revel A, Nathan Y, Bor A, Gacitua H (2005). Oocyte recovery, embryo development and ovarian function after cryopreservation and transplantation of whole sheep ovary.. Hum Reprod.

[35] Gavish Z, Ben Haim M, Arav A (2008). Cryopreservation of a whole liver.. Rejuvenation Res.

[36] O'Brien JK, Robeck TR (2006). Development of sperm sexing and associated assisted reproductive technology for sex preselection of captive bottlenose dolphins (Tursiops truncatus).. Reprod Fertil Dev.

[37] Si W, Hildebrandt TB, Reid C, Krieg R, Ji W (2006). The successful double cryopreservation of rabbit (Oryctolagus cuniculus) semen in large volume using the directional freezing technique with reduced concentration of cryoprotectants.. Theriogenology.

[38] Fujiki H, Suganuma M, Okabe S, Sueoka E, Suga K (1999). Mechanistic findings of green tea as cancer preventive for humans.. Proc Soc Exp Biol Med.

[39] Yamaguchi K, Honda M, Ikigai H, Hara Y, Shimamura T (2002). Inhibitory effects of (-)-epigallocatechin gallate on the life cycle of human immunodeficiency virus type 1 (HIV-1).. Antiviral Res.

[40] Yanagawa Y, Yamamoto Y, Hara Y, Shimamura T (2003). A combination effect of epigallocatechin gallate, a major compound of green tea catechins, with antibiotics on Helicobacter pylori growth in vitro.. Curr Microbiol.

[41] Lanping M, Zaiqun L, Bo Z, Li Y, Zhongli L (2000). Inhibition of free radical induced oxidative hemolysis of red blood cells by green tea polyphenols.. Chinese Sci Bull.

[42] Roth Z, Aroyo A, Yavin S, Arav A (2008). The antioxidant epigallocatechin gallate (EGCG) moderated the deleterious effects of maternal hypothermia on follicle-enclosed oocytes in mice.. Theriogenology.

[43] Kumazawa S, Kajiya K, Naito A, Saito H, Tuzi S (2004). Direct evidence of interaction of a green tea polyphenol, epigallocatechin gallate, with lipid bilayers by solid-state Nuclear Magnetic Resonance.. Biosci Biotechnol Biochem.

[44] Salvucci O, Yao L, Villalba S, Sajewicz A, Pittaluga S, Tosato G (2002). Regulation of endothelial cell branching morphogenesis by endogenous chemokine stromal-derived factor-1.. Blood.

[45] Crowe LM, Crowe JH (1988). Trehalose and dry dipalmitoylphosphatidylcholine revisited.. Biochim Biophys Acta.

[46] Wolkers WF, Tablin F, Crowe JH (2002). From anhydrobiosis to freeze-drying of eukaryotic cells.. Comp Biochem Physiol A Mol Integr Physiol.

[47] Crowe LM, Crowe JH, Rudolph A, Womersley C, Appel L (1985). Preservation of freeze-dried liposomes by trehalose.. Arch Biochem Biophys.

[48] Mazur P (1963). Kinetics of water loss from cells at subzero temperatures and the likelihood of intracellular freezing.. J Gen Physiol.

[49] Ma X, Jamil K, Macrae TH, Clegg JS, Russell JM, Villeneuve TS, Euloth M, Sun Y, Crowe JH, Tablin F, Oliver AE (2005). A small stress protein acts synergistically with trehalose to confer desiccation tolerance on mammalian cells.. Cryobiology.

[50] Han DW, Matsumura K, Kim B, Hyon SH (2008). Time-dependent intracellular trafficking of FITC-conjugated epigallocatechin-3-O-gallate in L-929 cells.. Bioorg Med Chem.

[51] Rindler V, Luneberger S, Schwindke P, Heschel I, Rau G (1999). Freeze drying of red blood cells at ultra low temperatures.. Cryobiology.

[52] Abdelwahed W, Degobert G, Fessi H (2006). Freeze-drying of nanocapsules: impact of annealing on the drying process.. Int J Pharm.

[53] Searles JA, Carpenter JF, Randolph TW (2001). Annealing to optimize the primary drying rate, reduce freezing-induced drying rate heterogeneity, and determine T(g)' in pharmaceutical lyophilization.. J Pharm Sci.

[54] Mazur P, Rall WF, Rigopoulos N (1981). Relative contributions of the fraction of unfrozen water and of salt concentration to the survival of slowly frozen human erythrocytes.. Biophys J.

[55] Hubel A, Cravalho EG, Nunner B, Körber C (1992). Survival of directionally solidified B-lymphoblasts under various crystal growth conditions.. Cryobiology.

[56] Crowe JH, Crowe LM, Wolkers WF, Oliver AE, Ma X (2005). Stabilization of dry mammalian cells: lessons from nature.. Integ Comp Biol.

[57] Crowe JH, Leslie SB, Crowe LM (1994). Is vitrification sufficient to preserve liposomes during freeze-drying?. Cryobiology.

[58] Thephinlap C, Ounjaijean S, Khansuwan U, Fucharoen S, Porter JB, Srichairatanakool S (2007). Epigallocatechin-3-gallate and epicatechin-3-gallate from green tea decrease plasma non-transferrin bound iron and erythrocyte oxidative stress.. Med Chem.

[59] Tipoe GL, Leung TM, Hung MW, Fung ML (2007). Green tea polyphenols as an anti-oxidant and anti-inflammatory agent for cardiovascular protection.. Cardiovasc Hematol Disord Drug Targets.

[60] Mazur P, Cole KW (1985). Influence of cell concentration on the contribution of unfrozen fraction and salt concentration to the survival of slowly frozen human erythrocytes.. Cryobiology.

[61] De Loecker W, Kopelov VA, Grischenko VI, De Loecker P (1998). Effects of cell concentration on viability and metabolic activity during cryopreservation.. Cryobiology.

[62] Kusadasi N, Van Soest PL, Mayen AE, Koevoet JLM, Ploemacher RE (2000). Successful short-term ex vivo expansion of NOD/SCID repopulating ability and CAFC week 6 from umbilical cord blood.. Leukemia.

[63] Wu D, Guo Z, Ren Z, Meydani SN (2007). Green tea catechin EGCG suppresses T cell mediated function through inhibiting cell division and reducing cell survival.. FASEB J.

[64] Hamza A, Zhan CG (2006). How can -(-) Epigallocatechin gallate from green tea prevent HIV-1 infection? Mechanistic insights from computational modeling and the implication for rational design of anti HIV-1 entry inhibitors.. J Phys Chem.

[65] Kawai K, Tsuno NH, Kitayama J, Okaji Y, Yazawa K (2004). Epigallocatechin gallate attenuates adhesion and migration of CD8+ T cells by binding to CD11b.. J Allergy Clin Immunol.

[66] Rindler V, Heschel I, Rau G (1999). Freeze-drying of red blood cells: how useful are freeze/thaw experiments for optimization of the cooling rate?. Cryobiology.

[67] Crowe LM, Reid DS, Crowe JH (1996). Is trehalose special for preserving dry materials?. Biophy J.

